# Modeling and Forecasting the GPS Zenith Troposphere Delay in West Antarctica Based on Different Blind Source Separation Methods and Deep Learning

**DOI:** 10.3390/s20082343

**Published:** 2020-04-20

**Authors:** Qingchuan Zhang, Fei Li, Shengkai Zhang, Wenhao Li

**Affiliations:** Chinese Antarctic Center of Surveying and Mapping, Wuhan University, 129 Luoyu Road, Wuhan 430079, China; zqc@whu.edu.cn (Q.Z.); zskai@whu.edu.cn (S.Z.); wh_li@whu.edu.cn (W.L.)

**Keywords:** GPS, ZTD, ICA, PCA, deep learning

## Abstract

Tropospheric delay is an important error source in global positioning systems (GPS), and the water vapor retrieved from the tropospheric delay is widely used in meteorological research such as climate analysis and weather forecasting. Most zenith tropospheric delay (ZTD) models are presently used as positioning corrections, and few models are used for the estimation of water vapor, especially in Antarctica. Through two blind source separation algorithms (principal component analysis (PCA) and independent component analysis (ICA)), a back-propagation (BP) neural network and a deep learning technique (long short-term memory (LSTM) network), we establish an hourly high-accuracy ZTD model for GPS meteorology using the GPS-ZTD from 52 GPS stations in West Antarctica. Our results show that under the condition in which the principal components (PCs) and independent components (ICs) remain fixed after decomposition, the mean accuracy of the models for West Antarctica using PCA or ICA are better than 10 mm. Compared with the ZTDs from the nonmodeling stations, the mean root mean square (RMS) of the PCA and ICA models are 9.3 and 8.9 mm, respectively, and the correlation coefficients between the GPS-ZTD and model-ZTDs all exceed 90%. The accuracy of the ICA model is slightly higher than that of the PCA model, and the ICs of the ICA model show more consistent spatial responses. The six-hour forecast is the best among the forecast results, with a mean correlation coefficient of 90.6% and a mean RMS of 7.2 mm using GPS-ZTD. The long-term forecast result is significantly inaccurate, as the correlation coefficient between the 24-h forecast and GPS-ZTD is only 63.2%. Generally modest results have been achieved (HSS ≤ 0.38). Furthermore, the forecast accuracy in coastal areas is lower than that in inland areas. Our study confirms that the combined use of ICA and deep learning in ZTD modeling can effectively restore the original signals, and short-term forecasting can be effectively used in GPS meteorology. However, further development of the technology is necessary.

## 1. Introduction

GPS signals are delayed when passing through the troposphere of the Earth [1]. As one of the main error sources of GPS positioning, such delays are also a key consideration in GPS meteorology [2]. The change in the zenith tropospheric delay (ZTD) is closely related to the pressure and the temperature around the station, and the most important greenhouse gas in atmospheric water vapor can be derived by using the ZTD estimates from GPS (GPS-ZTD) [3]. Numerous highly accurate ZTD data are used as supplements in meteorological research, and these data are of great significance to Antarctica, which lacks meteorological data. The number of GPS stations in Antarctica has increased in recent years with the continuous development of POLENET projects [4]. However, the current distribution of GPS stations remains sparse and not all of the stations are continuously operated, which limits the ability to obtain sufficient GPS-ZTD data over a large area. One solution is to use ZTD models.

Considering that the meteorological stations in Antarctica are sparse, this study focuses on a ZTD model without in situ meteorological data. Combining meteorological models with classic ZTD models, such as those of Saastamoinen [5] and Hopfield [6], is commonly used as a ZTD modeling method when in situ meteorological data are difficult to collect. The main representative models are the University of New Brunswick (UNB) series models [7,8,9], the Global Pressure/Temperature (GPT) series models [10,11,12], the Improved Tropospheric Grid (ITG) models [13], and the Chinese Tropospheric Model (Ctrop) model [14]. With the continuous updating of reanalysis data in meteorological modeling, the accuracy and reliability of combined ZTD models can be further enhanced. In addition, increased attention has been paid to the method of directly modeling ZTDs. The direct modeling method requires fewer parameters, entails an easier and clearer modeling process, and does not require the downloading and processing of large amounts of numerical weather data. Models using the direct method include the Global Zenith Tropospheric Delay (GZTD) series models [15,16,17], the SHAO-H models [18], the IGGtrop series models [19,20,21], and the Global Empirical Orthogonal Function Troposphere (GEOFT) models [22]. The key consideration in the direct modeling method is to derive accurate spatiotemporal features about ZTDs. However, although many ZTD models exist, most are empirical models for positioning correction and have low temporal resolution (daily or above), and many limitations arise in applications of high-resolution and high-accuracy estimations of water vapor. Therefore, studies focusing on the direct modeling ZTD suitable for GPS meteorology are worthy of attention.

Deep learning originated from artificial intelligence networks and was proposed by Hinton et al. [23]. By building a layered structure similar to the human brain, this method can gradually extract features of the input data from the bottom layer to the top layer to build a mapping relationship between underlying signals and high-level semantics [24]. Suparta and Alhasa successfully used a method called the adaptive neuro fuzzy inference system (ANFIS) to establish a ZTD model based on a single GPS station [25]. Xiao et al. established a real-time regional ZTD model in Japan using a back-propagation (BP) neural network [26]. However, the aforementioned studies did not consider the features of large-scale space and continuous time. To simultaneously derive the spatiotemporal features of the Antarctic ZTD, we used the so-called blind source separation (BSS) algorithm, which can extract the source signals from mixed signals. The main purpose of the BSS algorithm is to determine the source signals from the observation for physical interpretation by minimizing the number of assumptions and exploring the most reasonable options. Many applications can be pursued in geoscience studies, especially for GPS time series analyses [27]. The main methods for BSS are principal component analysis (PCA) and independent component analysis (ICA), and both have been widely used in the spatiotemporal filtering of GPS time series [28,29,30]. The BSSs are regarded as convenient and practical ZTD modeling methods, particularly for modeling spatiotemporal features, where the direct processing of high-dimensional raw data can be avoided, the efficiency of data processing can be improved, and the spatiotemporal features of data can be better understood. Dai et al. [31] established empirical ZTD models in China and the United Kingdom using ICA, and their models with centimeter accuracy outperformed the UNB3 and GPT2w models. However, their time resolution is only one day, but the present ZTD model requires a higher time resolution when used in GPS water vapor applications. In our study, we improved the time resolution to one hour and used PCA/ICA for data processing and analysis. During the modeling phase, we chose to maintain the time information (components) and use the neural network to model the spatial information (spatial response of components). Then, by combining the time and space information, we derived a high-resolution ZTD model. Finally, we used the long short-term memory (LSTM) network [32] to train the separated components to realize ZTD forecasting.

By taking the Transantarctic Mountains as the dividing boundary, we split Antarctica into West Antarctica and East Antarctica (http://wikipedia.moesalih.com/Antarctica). There are few GPS stations in East Antarctica, and most are located in coastal areas and have low spatial resolution. In contrast, the spatial resolutions of the GPS stations in West Antarctica are relatively high. Therefore, based on the ZTDs derived from 52 GPS stations in West Antarctica from 2014 to 2018, a BP neural network, and deep learning technique (LSTM network), we used PCA/ICA and BP neural networks to build a high-accuracy ZTD model and analyze the differences between PCA and ICA in the separation of source signals. The ZTD forecast is realized using the LSTM network, and the corresponding results are analyzed. The structure of this study is as follows. The methods and data are described in Section 2. The ZTD modeling process is described in Section 3. The model evaluation is discussed in Section 4. The conclusion is presented in Section 5.

## 2. Methods and Data

### 2.1. PCA

PCA, also known as empirical orthogonal functions, is a widely used feature extraction algorithm. With singular value decomposition, the covariance matrix of the data can be transformed into a set of orthogonal eigenvectors with corresponding principal components (PCs). The first PC is used to explain the direction of the maximum variance in the data, the second PC is used to find the direction of the second variance that is orthogonal to the first, and so on. Through this process, the data are projected based on the definitions of PCs [33,34].

By assuming a regional network of a GPS-ZTD time series with *n* stations and *m* hours, the (*m* × *n*) real-valued matrix $X\left(t_{i},x_{j}\right)$ (*i* = 1,2…*m*; *j* = 1,2…*n*) can be constructed. Each column of **X** represents the demeaned and detrended ZTDs from a single station, and each row of **X** represents the ZTDs for all *n* stations in a single epoch. The element of covariance matrix **B** is defined as follows:

(1) $$\;\;b_{ij}=\frac{1}{m-1}\sum_{k=1}^{m}X\left(t_{k},x_{i}\right)X\left(t_{k},x_{j}\right)$$

Then, **B** can be decomposed as follows:

(2) $$B=V\Lambda V^{T}$$

where the **Λ** matrix has k nonzero diagonal eigenvalues (*n* ≥ k), and the eigenvector matrix $V^{T}$ is the (*n* × *n*) matrix with orthonormal rows. Thus, the orthonormal function basis **V** can be expanded to **X** as follows:

(3) $$X\left(t_{i},x_{j}\right)=\sum_{k=1}^{n}a_{k}\left(t_{i}\right)v_{k}\left(x_{j}\right)$$

where $a_{k}\left(t_{i}\right)$ is derived by the following equation:

(4) $$\;a_{k}\left(t_{i}\right)=\sum_{k=1}^{n}\;X\left(t_{i},x_{j}\right)v_{k}\left(x_{j}\right)$$

where $a_{k}\left(t_{i}\right)$ is the kth PC of matrix **X**, and $v_{k}\left(x_{j}\right)$ is its corresponding eigenvector. To guarantee the data integrity as well as to compress data with redundant information (noise), the first several PCs with relatively large eigenvalues are kept, while the others are discarded. In general, the cumulative contribution of the remaining PCs should be higher than 95%. 

### 2.2. ICA

Although PCA is widely used, this method has its shortcomings [29]. The main reason is that PCA only considers the power and orthogonality of the components, and its components are unrelated, but the independence of different components is not considered. For example, two unrelated physical processes may also be statistically dependent. ICA is also a data-driven multivariate approach and belongs to the category of linear decompositions. Different from PCA, ICA is based on statistical independence and non-Gaussianity, and can be used to separate the independent components (ICs) from the mixed signal better. Based on ICA, the statistical dependence among the ICs of the analyzed signal can be minimized, and the essential structure of the source signal can be highlighted [35]. Therefore, when there is a dominant source or pattern in time series, PCA can work efficiently; when there are multiple competing sources, each PC may contain a mixed signal from different sources due to its simple assumption that each PC has an irrelevant relationship to others and the result of ICA is better. The main idea of ICA can be described as follows:

Assume that matrix **X** = [*x*_1_, *x*_2_, … *x*_M_], in which each column contains detrended and demeaned ZTDs from a single station and each row contains detrended and demeaned ZTDs for all stations in a single epoch, is generated by some underlying sources **S** = [ *s*_1_, *s*_2_, … *s*_N_]. Then, the ICA method can be described as follows:

**X** = **AS** (5)

where *A* represents the spatial responses. The assumptions for the ICA are as follows:

(1) *M* ≥ *N*;
(2) all source signals must be non-Gaussian; and
(3) each source **S***_i_* is mutually and statistically independent.

Then, the ICA finds a matrix **W** to transform the observation data into a random variable **Y***_i_*, in which **Y***_i_* is made to be as independent as possible.

**Y** = **WX** (6)

If **Y** is considered to be **S**, then **A** can be the inverse of **W**. In this study, we select the FastICA algorithm, as it is convergent, stable and fast [36]. 

After the PCA or ICA, ZTD time series are decomposed into temporal information and spatial information. The PCs or ICs represent temporal variations and their spatial responses represent spatial information. 

### 2.3. BP Neural Network

**Figure 1:**
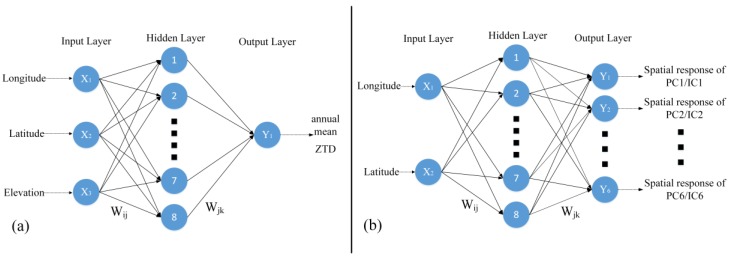
The topological structure of a neural network in this study. (**a**) Annual mean model; (**b**) spatial response model.

Artificial neural networks are algorithmic mathematical models that imitate the behavioral features of animal neural networks and carry out distributed parallel information processing. BP neural networks are a kind of multilayer feedforward neural network trained in error BP algorithms and have been used in many fields [37]. The topological structure of a neural network is shown as follows:

The input layer of the nodes in each layer in Figure 1 can be calculated as follows:

(7) $${\mathrm{LayerIn}}_{n,j}=\sum_{j=1}^{n}W_{i,j}X_{i}+\theta_{j}$$

where *n* represents the number of network layers, $W_{i,j}$ and $\theta_{j}$ represent the weight and threshold of the *n*th layer, $X_{i}$ is the corresponding input layer, and $LayerOut_{n,i}$ is the intermediate parameter of the model. The corresponding output layer is expressed as follows:

(8) $$LayerOut_{n,i}=\frac{1}{1+\exp\left(-LayerIn_{n,j}\right)}=\frac{1}{1+\exp\left(-\sum_{j=1}^{n}W_{i,j}X_{i}-\theta_{j}\right)}$$

where $LayerOut_{n,i}$ is the calculated output. According to the gradient descent algorithm, the error can be calculated by comparing the network’s estimated value $LayerOut_{n,i}$ with the observed value $Y_{i}$. Then, the model parameters are updated with the error BP as follows:

(9) φ(t+1)=φ(t)+ηδ(t)y(t)

where $\varphi =\left\{W_{i,j},\theta_{j}\right\}$, *y* is the neuron output, δ is the error, and η is the learning efficiency. 

In this paper, we used the BP neural network to model the annual mean ZTD and spatial response of PCs/ICs. In the annual mean model, the longitude, latitude, and elevation of the station were used as the input data and the annual mean ZTD of the station was used as the target. In addition, the annual mean ZTD of the station didn’t depend on the time. In the spatial response model, the longitude and latitude of stations were used as the input data and the spatial response of all PCs/ICs were used as the target. For two models, 70% of samples were used for training and 30% of samples were used for validation and testing. We set eight hidden neurons to two models, and the Levenberg–Marquardt algorithm was used.

### 2.4. LSTM Network

**Figure 2:**
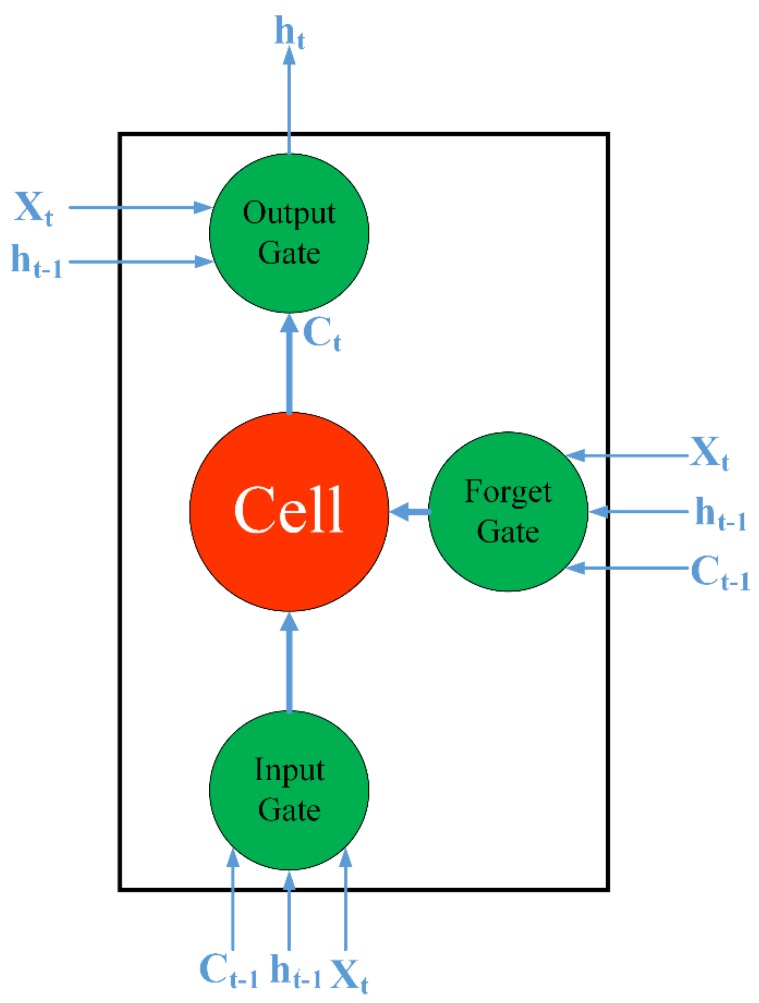
The structural diagram of the long short-term memory (LSTM) unit.

The LSTM network is a kind of recurrent neural network (RNN). By using memory cells and gating units to control the information flow, the LSTM network can prevent the gradient from vanishing rapidly; this scenario is a critical problem in RNNs and overcoming this problem can improve the RNN’s ability to process long-term sequences [32]. The structural diagram of the LSTM unit at time t is as follows:

The input gate in Figure 2 decides whether new information can enter the current memory unit, the forget gate decides whether to retain the historical information stored in the current memory unit, and the output gate decides whether to pass the latest cell output to the next layer. The forward calculation process is given by the following equations:

(10) $$i_{t}=\sigma \left(W_{it}x_{t}+W_{ir}h_{t-1}+W_{ic}c_{t-1}+b_{i}\right)$$

(11) $$f_{t}=\sigma \left(W_{ft}x_{t}+W_{fr}h_{t-1}+W_{fc}c_{t-1}+b_{f}\right)$$

(12) $$C_{t}=f_{t}C_{t-1}+i_{t}g\left(W_{cx}x_{t}+W_{cr}h_{t-1}+b_{c}\right)$$

(13) $$o_{t}=\sigma \left(W_{ot}x_{t}+W_{or}h_{t-1}+W_{oc}c_{t}+b_{o}\right)$$

where *t* is the current time; $i_{t}$, $f_{t}$, and $o_{t}$ are the values of the input gate, forget gate, and output gate, respectively; $C_{t}$ is the state of the current time’s memory cell; $h_{t-1}$ is the output state of the previous hidden layer; and *σ* is the activation function of each node in the network, in which the sigmoid function is used. 

In this paper, a sequence input layer inputs the temporal PC/IC data into the network. We set the LSTM layer to 200 hidden units, set the solver to “adam”, and conducted 250 epochs. To prevent a gradient explosion, we set the gradient threshold to 1. The initial learning rate was 0.005, which can be reduced by multiplying the factor by 0.2 after 125 rounds of training. After implementing this setup, we trained the components obtained from the PCA/ICA.

### 2.5. Data

After more than 30 years of development, GPS technology has become an important tool for geodesy and geophysics and has been widely used in monitoring the earth’s various processes on land, water, ice, and in the atmosphere [38]. To improve the utilization of GPS data, the Nevada Geodetic Laboratory (NGL) collected and processed the GPS data from more than 17,000 stations around the world. All of the data products, including the ZTD estimates, are available online with the associated processing strategies.

**Figure 3:**
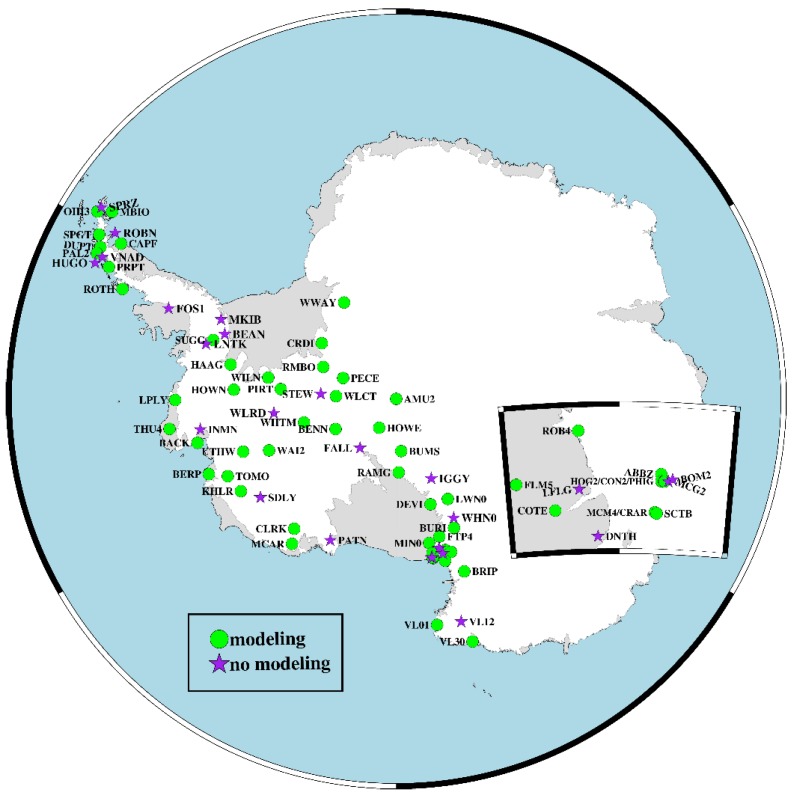
Distribution of modeling and nonmodeling stations in West Antarctica.

There are 204 stations in Antarctica according to NGL, including 186 stations in the western part, so we chose West Antarctica as the research area. The 186 stations in West Antarctica are not continuously operated; the observation time varies from several days to 22 years. In selecting suitable stations to be the modeling station, we first merged stations with the same locations. Then, we counted the number of stations with missing data (stations with rates of less than 0.3) every five years, from 2000 to 2018, e.g., 2000–2004, 2001–2005, 2002–2007… 2014–2018). According to the results, the largest number of eligible stations is from 2014 to 2018, with a total number of 57 stations. In consideration of the spatial distribution, we selected 52 stations as the modeling stations for the period between 2014 and 2018. Simultaneously, the 21 stations that were not included in the modeling process were used to validate the model and we called these stations the nonmodeling stations (Figure 3).

## 3. Process of ZTD Modeling

### 3.1. Data Preprocessing

**Figure 4:**
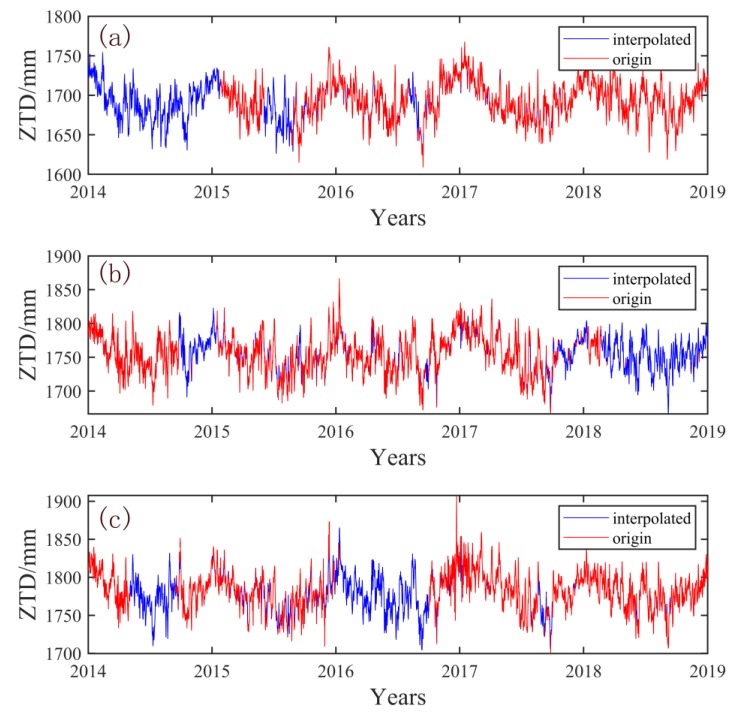
Zenith tropospheric delay (ZTD) time series before and after interpolation at the (**a**) HOG2, (**b**) KHLR, and (**c**) WAI2 stations, with missing rates of 29.5%, 30%, and 28.6%, respectively.

Due to the continuity requirement of PCA/ICA, the missing data need to be filled initially. The method is structured as follows. First, the ZTD residual time series is obtained by removing the linear and periodic terms as well as the outliers from the original ZTD time series. Then, a widely used meteorology interpolation method called the regularized expectation maximization (RegEM) algorithm is used to compute the missing data in the ZTD residual time series. In the RegEM algorithm, ridge regression is used to realize regression regularization and parameter estimation, and a cross test is used to realize spatiotemporal matrix interpolation within a given accuracy [39]. The RegEM algorithm can estimate the missing data with its simultaneous and diachronic covariance matrix, which could be used to analyze its spatial covariance, stationary time covariance, or periodic stationary time covariance. Furthermore, the RegEM algorithm neither relies on external mathematical models nor introduces external prior information; instead, the algorithm performs an interpolation according to the physical background of the data and the correlation between stations, which makes it a good interpolation method for time series with a few missing data (for model details, see Figure S1 and Table in Supplementary Material). Finally, the previously removed linear and periodic terms are added back to the interpolated residuals to obtain the continuous ZTD time series. Figure 4 shows the data interpolation results of three stations with the largest missing rates. The results of the RegEM interpolation can keep the main features in the original data. Considering that the GPS-ZTD data time resolution is 5 min, we took the hourly median ZTD to resample its time resolution to 1 h.

### 3.2. ZTD Modeling

The ZTD modeling steps, in which the ICA method is taken as an example, are as follows.

#### 3.2.1. Building the Annual Mean Model

Before the PCA/ICA is used to process the data, the mean value of the data needs to be removed. When we build the model, we must restore its mean to obtain the correct ZTD. Therefore, a stable annual mean model of the ZTD should be built initially. First, the annual mean ZTD for each station is calculated. Then, we use the BP neural network to train and build the annual mean model. We set the input layer to ‘longitude, latitude, and elevation of the station’, and set the output layer to ‘annual mean ZTD’. Eight hidden neurons are selected. The gradient descent learning algorithm is used to adjust the error according to the least-square criterion. The Levenberg–Marquardt algorithm was used in this paper.

#### 3.2.2. Choosing the Appropriate Number of ICs

It is essential to choose an appropriate number of components for ICA. Selecting too many components will introduce an excessive noise source, whereas too few components will mix the source signal. Miller et al. used Horn’s parallel analysis to select the appropriate number of components before ICA processing [40]. As one of the methods to determine the number of factors retained, Horn’s parallel analysis compares the mean value or the 95th percentile of the eigenvalues of a real data matrix with a random data matrix to decide whether the factor is retainable [41]. The result of Horn’s parallel analysis shows that six components are appropriate for our research. Then, the ZTD time series after de-meaning and whitening are decomposed by ICA to obtain the spatial information matrix and the time information matrix.

#### 3.2.3. Building the Spatial Response Model

**Figure 5:**
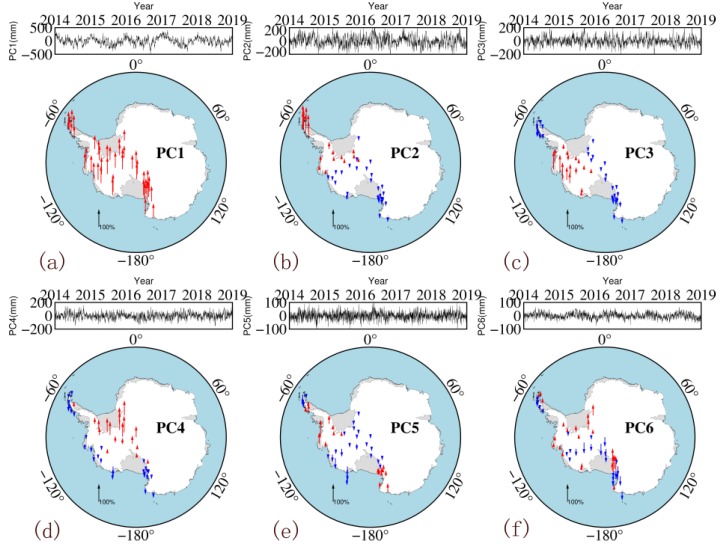
Six principal components (PC) of principal component analysis (PCA) solution. From (**a**–**f**) are PC1, PC2, PC3, PC4, PC5 and PC6, respectively. (The time series at the top of each panel shows the PC of each component; the maps at the bottom show the normalized spatial responses (red: positive response; blue: negative response)).

**Figure 6:**
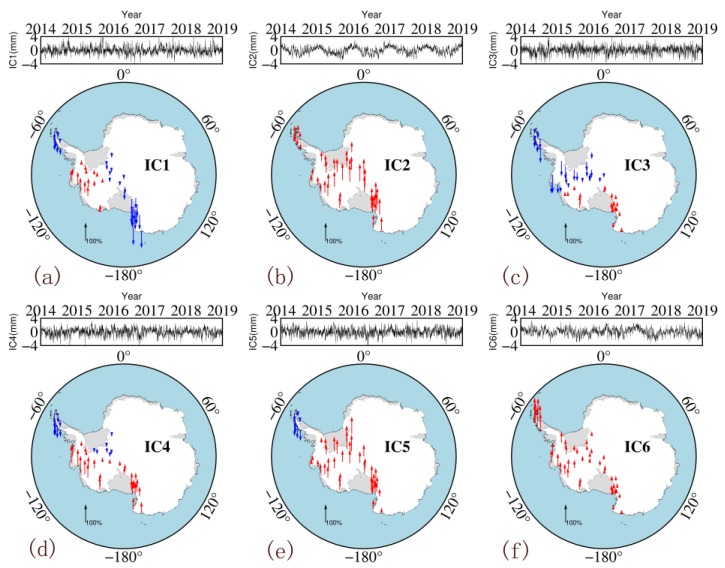
Six independent components (IC) of independent component analysis (ICA) solution. From (**a**–**f**) are IC1, IC2, IC3, IC4, IC5 and IC6, respectively. (The time series at the top of each panel shows the IC of each component; the maps at the bottom show the normalized spatial responses (red: positive response; blue: negative response)).

After PCA/ICA processing, the data matrix is decomposed into two parts: the spatial response matrix (spatial information) and the component matrix (time information). Figure 5 and Figure 6 show the six PCs and six ICs and their normalized spatial responses. The spatial responses obtained by the two methods entail certain spatial characteristics. The four PCs in the PCA results (PC2, PC4, PC5 and PC6) all have stations with spatial responses that are the opposite of those in their surroundings, indicating their low spatial consistency. The spatial response obtained by the ICA shows the same trend within an area, indicating that its spatial consistency is better than that of PCA. Similar to the annual mean model, the BP neural network is also used, in which the input is the longitude and latitude of the station, whereas the output is the spatial response corresponding to different components.

The PCs extracted by the PCA method have no clear physical meaning but do have a mixed signal from different sources. PC1 accounts for 54.14% of the total signal energy, whereas the first three components account for more than 85% of the total signal energy. By contrast, the ICs are mutually independent, with each IC representing a single signal. For example, IC2 is mainly an annual periodic signal, and the change in the IC6 signal in 2017 corresponds to the El Niño event of that year. Further research on the different ICs can help to comprehensively explain the impact of different physical factors on ZTDs. As the purpose of this study is to build a high-accuracy and high-resolution ZTD model that can be used in GPS meteorology, the analysis of the aforementioned relationship between ICs and potential physical resources will be carried out in the future.

#### 3.2.4. Obtaining the ZTD Models

**Figure 7:**
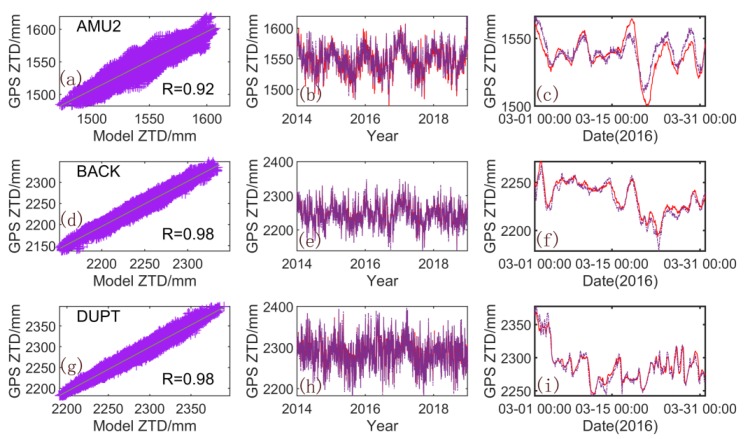
Comparison of GPS-ZTD and PCA-ZTD for the AMU2, BACK, and DUPT stations. (**a**,**d**,**g**) are correlation between the GPS-ZTD and PCA-ZTD; (**b**,**e**,**h**) are their time series from 2014 to 2018; (**c**,**f**,**i**) are time series in one month. Red solid line: PCA-ZTD; purple solid line: GPS-ZTD.).

**Figure 8:**
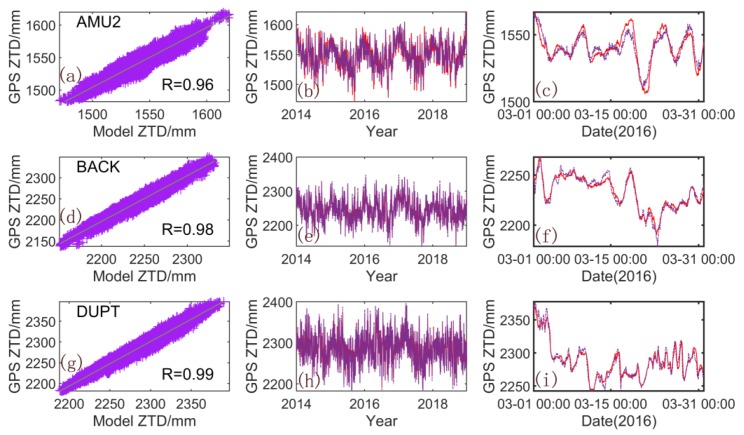
Comparison of GPS-ZTD and ICA-ZTD for the AMU2, BACK, and DUPT stations. (**a**,**d**,**g**) are correlation between the GPS-ZTD and ICA-ZTD; (**b**,**e**,**h**) are their time series from 2014 to 2018; (**c**,**f**,**i**) are time series in one month. Red solid line: PCA-ZTD; purple solid line: GPS-ZTD.)

The annual mean model, the spatial response model, and the ICs are combined to obtain a ZTD model with the same timespan as that of the modeling data. The correlation among the GPS-ZTDs at three stations (AMU2, BACK, and DUPT) and that of the PCA-ZTD (ICA-ZTD), as well as their time series, are presented in Figure 7 (Figure 8). The correlation coefficients between the ZTD and models all exceed 0.9, with the modeling result of ICA slightly higher than that of PCA. For the time series, the five-year time series of the ICA models are consistent with the GPS-ZTD. For clarity, we selected one-month results for further analysis. The model established by using ICA can effectively simulate the change in GPS-ZTDs, especially for the AMU2 station.

ZTD forecasting is realized by introducing the LSTM network into the models. The components obtained from the LSTM network forecast are combined with the findings from the annual mean model and the spatial response model to realize the ZTD forecast for West Antarctica. The overall assessment of the two modeling methods (PCA and ICA) for West Antarctica is discussed in detail in Section 4.1 and Section 4.2.

## 4. Results and Discussion

### 4.1. Validation Using Modeling Station Data

**Figure 9:**
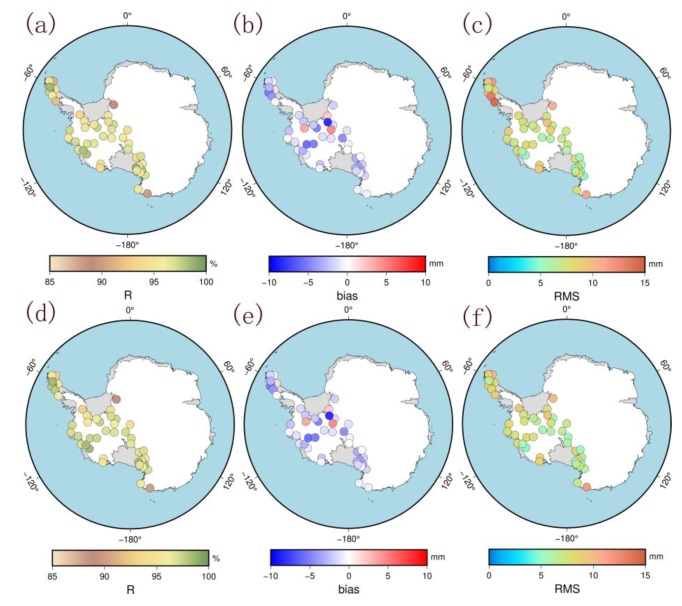
Distribution of the correlation coefficient R, bias, and RMS of the ZTD models and GPS-ZTD (**a**,**b**,**c**) are the result of PCA; (**d**,**e**,**f**) are the result of ICA).

In this section, we first used the ZTD data of the 52 modeling stations from 2014 to 2018 to evaluate the accuracy of the aforementioned models. We calculated the correlation coefficient R, bias, and root mean square (RMS) between the ZTD models and GPS-ZTD for each station. Table 1 shows the results for all of West Antarctica, the areas with elevations of less than 500 m, the areas with elevations greater than 500 m, and the Antarctic Peninsula. Figure 9 shows the spatial distributions of the coefficients R, bias, and RMS.

The PCA-ZTD or ICA-ZTD are strongly correlated with the GPS-ZTD. The accuracies of the two modeling methods are better than 10 mm, and the mean bias of the ICA modeling is the same as that for the PCA modeling. The mean correlation coefficient R (96.2%) and the mean RMS (7.1 mm) using ICA are slightly better than those using PCA (95.9% and 7.6 mm, respectively). The spatial distributions of the correlation coefficient R for the two models in Figure 9 are generally consistent and the bias and the RMS show some spatial characteristics, especially for the Antarctic Peninsula, whose values are significantly different from those of the other regions. The Antarctic Peninsula is far from the Antarctic continent and the water vapor is rich and changes rapidly, which leads to greater changes in the ZTD in this region than in other regions, so its RMS is higher than other regions. We calculated the mean correlation coefficient R, bias, and RMS of the stations located at elevations below 500 m and the stations located at elevations above 500 m (Table 1). When the elevation is less than 500 m, the mean bias and RMS are higher than those of the modeling stations above 500 m. In the validation of the results from the modeling stations, the accuracy of the two models for the stations at elevations less than 500 m is lower than that of stations at elevations greater than 500 m. This is because most of the stations at an elevation of less than 500 m are located in the coastal area, are rich in water vapor and show active troposphere change, which results in rapid changes in the ZTD. However, with increasing elevation, the atmosphere is thin, the water vapor content decreases, and the troposphere changes tend to be stable, which results in a decrease in the ZTD change and an increase in the accuracy of model simulation ZTD. The mean RMS from the ICA modeling is 7.7 mm, which is smaller than the 8.8 mm value from the PCA modeling. When the elevation is higher than 500 m, the mean bias and RMS of the two modeling methods are consistent. The results show that the accuracy of the ICA model is better than that of the PCA model in the areas where ZTD changes are more complex. This may be because the distribution of spatial responses of the independent components from ICA decomposition is more consistent and more suitable for modeling, so the model results are better. However, in the high-elevation area with relatively stable ZTD changes, the results of the two models show little difference. In addition, we calculated the results for the stations on the Antarctic Peninsula. The mean bias and RMS values of the two modeling methods are the highest, as shown in Table 1. The mean RMS from the ICA modeling is 8.3 mm and is significantly better than that from the PCA modeling (10.4 mm). This is also consistent with the previous description. In general, the accuracy of ICA is better than that of PCA based on validation using the modeling stations.

### 4.2. Validation Using Nonmodeling Station Data

**Figure 10:**
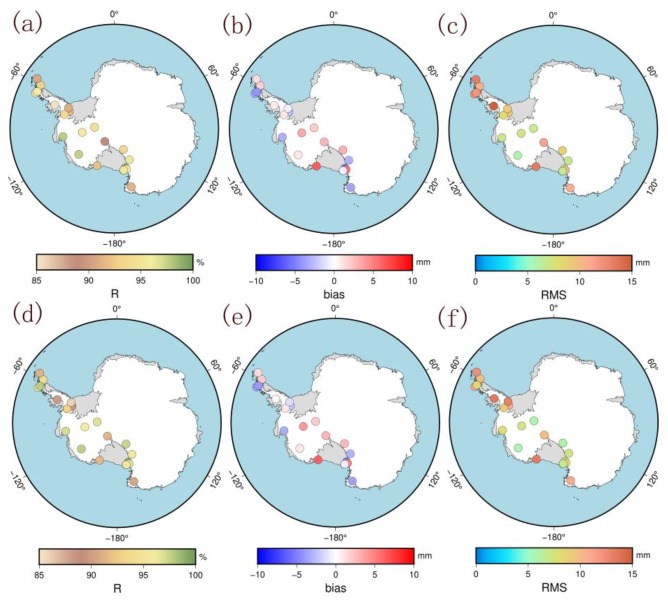
Distribution of correlation coefficient R, bias, and RMS between the ZTD models and GPS-ZTD of nonmodeling stations (**a**,**b**,**c**) are the result of PCA; (**d**,**e**,**f**) are the result of ICA).

The 21 GPS stations that did not participate in the modeling (called the nonmodeling stations) were selected to evaluate the accuracies of the two models. Table 2 shows the comparison of the correlation coefficient R, bias, and RMS of the two models with the GPS-ZTDs from the nonmodeling stations. Figure 10 shows the spatial distributions of the R, bias, and RMS values of each station.

The two ZTD models are all strongly correlated with the GPS-ZTD in Table 2 and Figure 10. In West Antarctica, the mean values of correlation coefficient R, bias, and RMS from the PCA model are 93.9%, 0.1 mm, and 9.3 mm, respectively. The results from the ICA model are slightly better than those from the PCA modeling, as its mean correlation coefficient, bias, and RMS are 94.1%, 0.0 mm, and 8.9 mm, respectively. The results show that the accuracies of the two modeling methods are better than 10 mm. For regional comparison, when the elevation is higher than 500 m, the mean RMS of the two modeling methods are lower than those of the stations in West Antarctica, and a negligible difference exists between the ICA and PCA modeling results. When the elevation is less than 500 m, the mean RMS of the two modeling methods are significantly higher than those of the stations in West Antarctica. The average RMS from the ICA model is 10.5 mm, which is lower than the 12.0 mm value from the PCA model; this result is also consistent with the results for the modeling stations. The main reason is that most of the stations with elevations lower than 500 m are located in coastal areas where water vapor is relatively abundant and changes rapidly, which increases the uncertainty in ZTD modeling. In contrast, most of the stations located in the inland areas have lower water vapor, indicating stable ZTD changes. As such, the simulation for inland areas is easier, and the results are better than those for coastal areas. In addition, when the elevation is less than 500 m, the results from the ICA model are better than those from the PCA model, whereas when the elevation is greater than 500 m, the difference is not significant between the two modeling methods. These findings indicate that both modeling methods can simulate the ZTDs in stable inland areas better that those in turbulent coastal areas. Meanwhile, for coastal areas with low elevations and abundant water vapor, the spatiotemporal feature modeling results extracted by the ICA are better than those extracted by the PCA. The findings further suggest that the feature extraction ability of the ICA is better than that of the PCA in complex environments. Then, the results for the Antarctic Peninsula are also calculated to further compare the PCA and ICA methods. Given that few modeling stations are located on the Antarctic Peninsula, especially in the southern part, the results for this region are validated to further compare PCA and ICA. The results show that the mean RMS from the ICA model is 10.9 mm, which is slightly better than that from the PCA model. This result also shows that the ICA is more interpretable and reliable in extracting spatiotemporal features than the PCA.

Comparison of the ZTD models and GPS-ZTD from modeling and nonmodeling stations shows that the accuracies of the two modeling methods are better than 10 mm for West Antarctica. The results from the ICA model are slightly better than those from the PCA model, as the ICA can better capture the real changes in ZTDs in most cases. These findings indicate that the ICA can be used in water vapor estimations and other meteorological applications.

### 4.3. Validation of ZTD Forecasting

With the development of GPS, ZTD data have been increasingly applied in many meteorological studies, such as weather forecasting [42]. Each component obtained from the BSS in this study is trained using the LSTM network to realize a regional ZTD forecast. The ICA modeling results, which are slightly better than the PCA modeling results, are used in the following experiments. We use the ICs from 1 January 2014 to 31 December 2017 (57 stations total) as the training data and set three forecast timespans of 6, 12, and 24 h. Then, the forecast results of 31 days (1–31 January 2018) were obtained and compared with the GPS-ZTDs. Finally, the forecast results of the different timespans were compared and analyzed.

**Figure 11:**
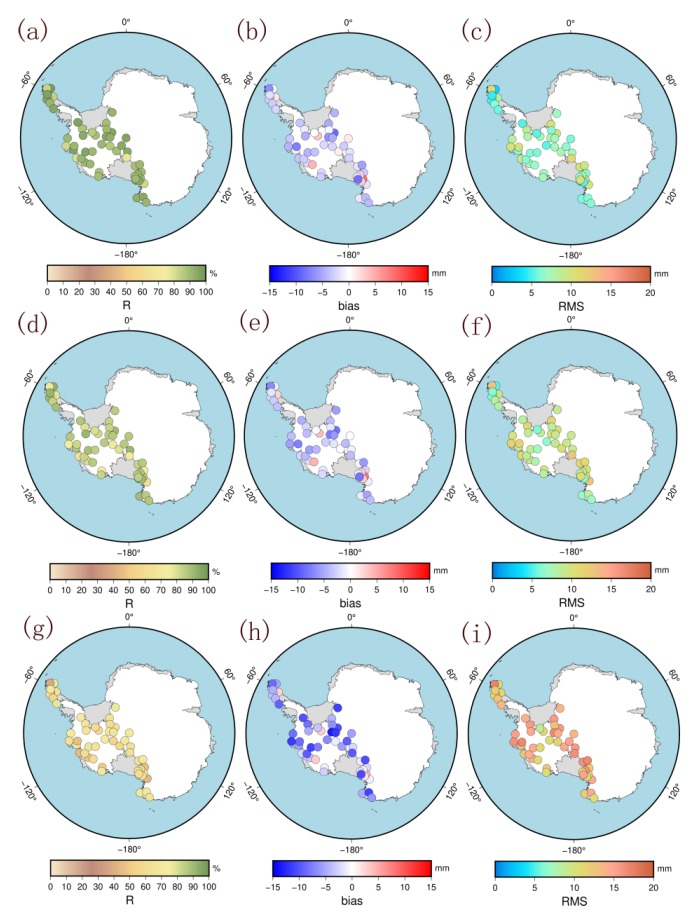
Distribution of correlation coefficient R, bias, and RMS between the forecasting results and GPS-ZTD (**a**,**d**,**g**) are six-hour forecast results; (**b**,**e**,**h**) are 12 h forecast results; (**c**,**f**,**i**) are 24 h forecast results).

**Figure 12:**
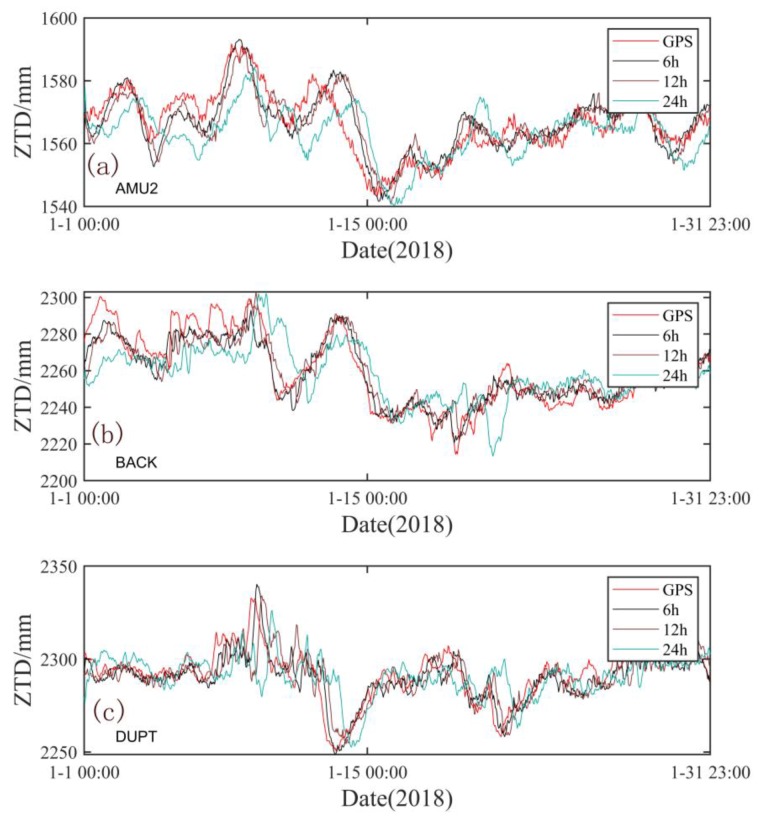
Comparison of forecasted ZTDs and GPS-ZTDs at the (**a**) AMU2, (**b**) BACK, and (**c**) DUPT stations (black solid line: six-hour forecast result; brown solid line: 12 h forecast result; light blue solid line: 24 h forecast result).

Table 3 and Figure 11 show the statistical results and the spatial distribution of the correlation coefficient R, bias, and RMS between the forecasting results and GPS-ZTD, respectively, for the forecast timespans of 6, 12, and 24 h. The results show that the mean RMS of the stations at elevations less than 500 m are higher than those of the other areas for the three forecast timespans (6, 12, or 24 h), indicating that the forecast accuracy of the model for the low-elevation stations is lower than that for the high-elevation stations. Moreover, most of the stations at elevations less than 500 m are located in the coastal areas where water vapor is abundant and turbulent, so the forecast accuracy is not as good as that for the inland areas. Furthermore, the stations with the lowest accuracies are mainly located on the Antarctic Peninsula. When the forecast times are 6, 12, and 24 h, the mean RMSs of the stations on the Antarctic Peninsula are 7.6, 10.0, and 12.1 mm, respectively. These values are significantly higher than the mean values in West Antarctica and the mean values for all stations at elevations lower than 500 m. This finding can be attributed to the location of the Antarctic Peninsula, which is far from the Antarctic continent, and water vapor there is more abundant than in the other areas. The spatial features shown in Figure 11 are consistent with this description. With the extension of the forecast time, the correlation coefficient decreases but no obvious regional deviation can be observed. Moreover, the bias and RMS are significantly higher than those of the other stations in the coastal areas, especially on the Antarctic Peninsula. For further analysis, we used the time series of the forecasted ZTDs and GPS-ZTDs of AMU2, BACK, and DUPT from 0:00 on January 1 to 23:00 on January 31, 2018. The six-hour and 12-h forecasted ZTDs can basically reflect the real-time changes in the GPS-ZTDs. As can be seen from Figure 12, the six-hour forecasted ZTDs are in better agreement with the GPS-ZTDs whereas the 12-h forecasted ZTDs show some outliers. The 24-h forecasted ZTDs are not in good agreement with the GPS-ZTDs, indicating that accurate real-time ZTD changes cannot be retained. The accuracy of the ZTDs must be 10 mm or better for numerical meteorological model assimilation [43]; thus, the six-hour forecasted ZTDs can generally meet the requirements. However, in the future, we need to further optimize the algorithm to improve the forecast accuracy and timespan on the Antarctic Peninsula.

Finally, we calculated the Heidke Skill Score (HSS, range of −1 to 1, optimal skill is 1) of the forecast result with three different timespans (6, 12, or 24 h). The HSS is a measure of skill in forecasts and indicates the fraction of correct forecast after eliminating the random predictions [44]. In this study, we set HSS criterion for subsequent events:

(1) Event A. Rise ZTD: ZTD(*t* + 6 h) − ZTD(*t*) >= 0.
(2) Event non-A. Drop ZTD: ZTD(*t* + 6 h) − ZTD(*t*) < 0.

where *t* is time. The HSS can be calculated by Equation (14). Table 4 gives the definition of each element in Equation (14).

(14) HSS=2(ad−bc)/[(a+c)(c+d)+(a+b)(b+d)]

Here, *a* describes how many times event A occurred simultaneously in the observation and forecast; *b* is the number of times event A occurred in the forecast and event Non-A occurred in the observation; *c* is the number of times event Non-A occurred in the forecast and event A occurred in the observation; *d* is the number of times event Non-A occurred simultaneously in the observation and forecast.

The results show that when the forecast time is six hours, the model is the most skillful, with an HSS of 0.38, compared with 0.26 for the 12-h forecast result and 0.05 for the 24-h forecast result. The HSS of the 24-h forecast result is close to zero. This shows that when the forecast time is 24 h, the model has nearly no skill and the forecast ability is poor. This is also consistent with the previous conclusion.

## 5. Conclusions

In this study, two BSS algorithms, namely, PCA and ICA, were used to process the ZTD time series of 52 GPS stations in West Antarctica from 2014 to 2018. Combined with the BP neural network, two hourly ZTD models for GPS meteorology were established. Differences between PCA and ICA for ZTD modeling were compared and analyzed. Finally, the ZTD forecast was realized using the LSTM network. The GPS-ZTDs were used to validate the model. The results obtained in this study are as follows:

By using PCA or ICA for ZTD modeling, the mean accuracy for West Antarctica is better than 10 mm. The mean RMSs for the PCA and ICA models are 9.3 and 8.9 mm, respectively. The mean correlation coefficients between the two models and the GPS-ZTDs all exceed 0.9. The accuracy of the two models for the coastal areas is lower than those for the inland areas. In addition, the accuracy of the ICA model is better than that of the PCA model in the coastal areas, indicating that ICA is more accurate and suitable for modeling GPS-ZTD in West Antarctica.

Furthermore, by using the LSTM in ZTD forecasting, high-accuracy results can be achieved in a short time span. The mean correlation coefficient between the six-hour forecasted ZTDs and the actual GPS-ZTDs is 90.6%. However, when the forecast time was increased to 24 h, the correlation coefficient decreased to 63.2%. The RMS also increased from 7.2 to 13.3 mm. The forecast accuracy for stations in inland areas is higher than that in coastal areas. Generally modest results have been achieved (HSS ≤ 0.38).

The results of this study show that the combination of ICA and deep learning can be directly applied to high-accuracy regional modeling and forecasting of water vapor from GPS-ZTDs. Obtaining a better ZTD time series forecast that requires relatively longer data for modeling and deep learning could in turn improve the accuracy of forecasting. However, the distribution of GPS stations in Antarctica remains sparse. If more GPS stations are deployed in Antarctica, then more accurate ZTD spatiotemporal characteristics can be observed and modeled. By that time, the use of ICA and deep learning can be better applied to model the ZTD changes, and its relationship with different physical mechanisms can be further analyzed. The modeling method adopted in this study is expected to become a useful supplement for meteorological applications, such as climate analysis and weather forecasting, but further development of the technology is necessary.

## Figures and Tables

**Table 1:** Correlation coefficient R, bias, and root mean square (RMS) of the ZTD models and GPS-ZTD.

Model	West Antarctica			West Antarctica (*h* < 500 m)			West Antarctica (*h* ≥ 500 m)			Antarctic Peninsula		
	R/%	Bias/mm	RMS/mm	R/%	Bias/mm	RMS/mm	R/%	Bias/mm	RMS/mm	R/%	Bias/mm	RMS/mm
PCA	95.9	−1.5	7.6	95.9	−1.7	8.8	95.9	−1.4	6.9	95.4	−1.8	10.4
ICA	96.2	−1.5	7.1	96.5	−1.7	7.7	96.1	−1.4	6.8	96.6	−1.8	8.3

**Table 2:** Correlation coefficient R, bias, and RMS between the ZTD models and GPS-ZTD of nonmodeling stations.

Model	West Antarctica			West Antarctica (*h* < 500 m)			West Antarctica (*h* ≥ 500 m)			Antarctic Peninsula		
	R/%	Bias/mm	RMS/mm	R/%	Bias/mm	RMS/mm	R/%	Bias/mm	RMS/mm	R/%	Bias/mm	RMS/mm
PCA	93.9	0.1	9.3	92.1	0.7	12.0	94.8	−0.2	8.0	92.0	−0.7	11.2
ICA	94.1	0.0	8.9	93.5	0.5	10.5	94.5	−0.2	8.1	91.7	−0.8	10.9

**Table 3:** Correlation coefficient R, bias, and RMS between the forecasting results and GPS-ZTD.

Timespan	West Antarctica			West Antarctica (*h* < 500 m)			West Antarctica (*h* ≥ 500 m)			Antarctic Peninsula		
	R/%	Bias/mm	RMS/mm	R/%	Bias/mm	RMS/mm	R/%	Bias/mm	RMS/mm	R/%	Bias/mm	RMS/mm
6 h	90.6	−2.0	7.2	89.0	−0.5	7.7	91.3	−2.7	6.9	84.7	−2.0	7.6
12 h	83.0	−2.2	9.1	79.3	−0.3	10.0	84.9	−3.1	8.6	71.2	−1.2	10.0
24 h	63.2	−5.1	13.3	59.6	−2.4	13.5	65.1	−6.5	13.3	51.5	−0.9	12.1

**Table 4:** Heidke Skill Score Table.

Event Forecast	Event Observed	
	A	Non-A
A	*a*	*b*
Non-A	*c*	*d*

## Supplementary Materials

The following are available online at https://www.mdpi.com/1424-8220/20/8/2343/s1, Figure S1: The correlation and time series between the interpolated value and origin value at the AMU2 station. (Top: the missing rate is 10%; middle: the missing rate is 20%; bottom: the missing rate is 30%), Table S1: Correlation coefficient R, bias, and RMS between the interpolation result of RegEM and the original data at AMU2 during 2014–2018.
