# Supplementary material for: Modeling and Forecasting the GPS Zenith Troposphere Delay in West Antarctica Based on Different Blind Source Separation Methods and Deep Learning
Source: Sensors (Basel). 2020 Apr 20;20(8):2343. doi: 10.3390/s20082343 (PMC7219593; doi:10.3390/s20082343)
Supplement: Supplementary file 1 [file sensors-20-02343-s001.pdf]

Supplementary

Modeling and Forecasting the GPS Zenith Troposphere Delay in West Antarctica Based on Different Blind Source Separation Methods and Deep Learning

Qingchuan Zhang, Fei Li *, Shengkai Zhang and Wenhao Li

Chinese Antarctic Center of Surveying and Mapping, Wuhan University, 129 Luoyu Road, Wuhan 430079, China; zqc@whu.edu.cn (Q.Z.); zskai@whu.edu.cn (S.Z.); wh_li@whu.edu.cn (W.L.)

* Correspondence: fli@whu.edu.cn; Tel.: +86-27-6875-4269

1. RegEM

1.1. The Basic Principle of the RegEM Algorithm is as Follows:

Let X be a n×m ZTD matrix (the column vector contains the missing data), n is the number of epochs and m is the number of stations, generally meeting the requirement of n > m. The estimated mean and covariance matrices of X are $\mu \in R^{1\times m}$ and $\sigma \in R^{m\times m}$, respectively. The ZTD vector of all stations at a given epoch i is $X_i \in R^{1\times m}$, which has missing data. The ZTD vector composed of m_a stations for which the data are available at epoch i is $Xa \in R^{1\times ma}$, and the ZTD vector composed of m_m stations for which the data are missing at epoch i is $Xm \in R^{1\times mm}$. The mean values of Xa and Xm are $\mu_a \in R^{1\times ma}$ and $\mu_m \in R^{1\times mm}$, respectively. For a given epoch, the relationship between the vector with missing data and the vector with available data is modeled as follows:

$$X_m = \mu_m + (X_a - \mu_a)B + e$$

The matrix $B \in R^{Pa\times mm}$ is comprised of regression coefficients, and $e \in R^{1\times mm}$ is assumed to be a random vector with mean zero and unknown covariance matrix C. In each iteration of the algorithm, the mean μ and covariance matrix σ are given, and the conditional maximum likelihood estimates of the matrix of regression coefficients B and covariance matrix C of the residual are computed for each epoch with missing values. The missing data can be filled in using the following formula:

$$X'_m = \mu'_m + (x_a - \mu'_a)B'$$

μ'_m and μ'_a are the estimated values of the mean of the missing data and available data, respectively. B' is the estimated regression coefficient. After filling in the missing data, the new μ and σ are calculated. In each iteration, a k-fold cross validation (KCV) is used to adaptively select regularization parameters for each row of ZTD data.

1.2. Evaluation of Interpolation by RegEM

To verify the interpolation results, we used the ZTD residual time series of the AMU2 station with 99.51% data integrity as an example, and simulated the three missing rates of 10%, 20% and 30%. The interpolation result of RegEM was evaluated.

Table S1. Correlation coefficient R, bias, and RMS between the interpolation result of RegEM and the original data at AMU2 during 2014–2018.

Missing Rate / %	Correlation Coefficient / %	RMS / mm	bias / mm
10	97.7	4.3	−0.3
20	97.0	4.4	0.3
30	97.1	4.3	0.2

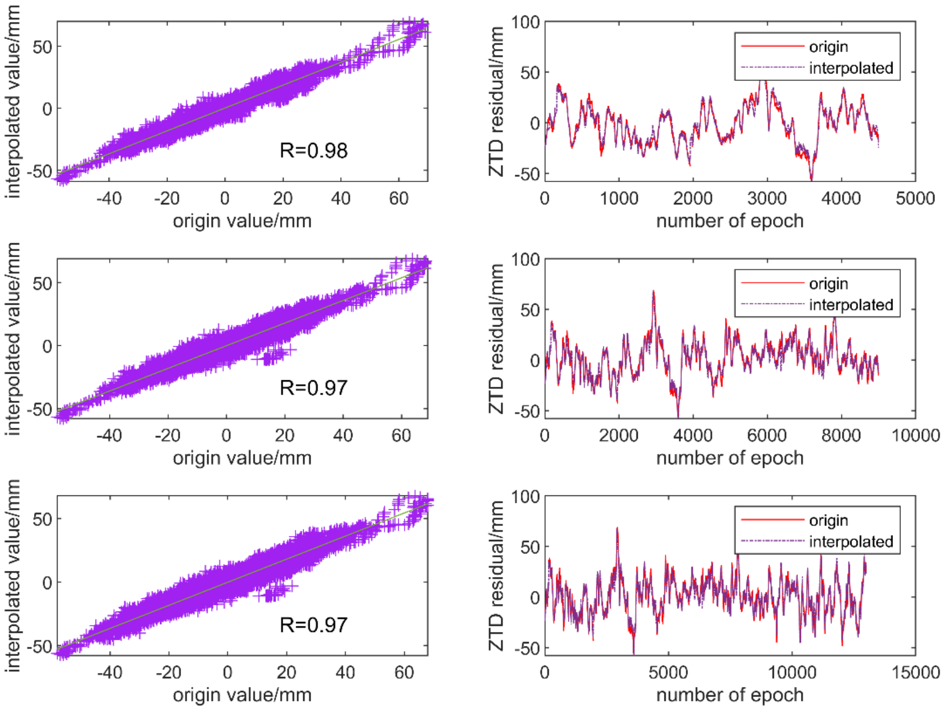

Figure S1. The correlation and time series between the interpolated value and origin value at the AMU2 station. (Top: the missing rate is 10%; middle: the missing rate is 20%; bottom: the missing rate is 30%).

Table S1 and Figure S1 show the comparison results between the interpolation value and the original value when the missing rate is 10%, 20% and 30%. In the three cases, the correlation coefficient between the interpolation value and the original value is approximately 97%, which means the correlation is very strong; the RMS and bias are 4.3 mm and 0.3 mm, respectively, in the three cases and both reach the mm level. The results of the three cases show no significant difference. This also shows that the interpolation results from the RegEM are in good agreement with the original results and can maintain a high interpolation accuracy at different missing rates (the highest in this paper is 30%). Therefore, the RegEM method is suitable for data interpolation in this study.
